# Developing an interprofessional people-centred care model for home-living older people with multimorbidities in a primary care health centre: A community-based study

**DOI:** 10.1016/j.rcsop.2022.100114

**Published:** 2022-02-05

**Authors:** Heini Kari, Hanna Kortejärvi, Raisa Laaksonen

**Affiliations:** a Division of Pharmacology and Pharmacotherapy, Faculty of Pharmacy, University of Helsinki, Viikinkaari 5 E (P.O.Box 56), 00014 Helsinki, Finland; b Division of Pharmaceutical Biosciences, Faculty of Pharmacy, Viikinkaari 5 E (P.O.Box 56), 00014, University of Helsinki, Helsinki, Finland

**Keywords:** People-centred care, Interprofessional collaboration, Clinical pharmacy, Primary healthcare, Older people, Participatory action research

## Abstract

**Background:**

The ageing population with multiple conditions and complex health needs has forced healthcare systems to rethink the optimal way of delivering services. Instead of trying to manage numerous diseases in a siloed approach, the emphasis should be on people-centred practice, in which healthcare services are tailored to people's needs and provided in partnership with them.

**Objective:**

The aim was to develop an interprofessional people-centred care model (PCCM), including the contribution of a clinically trained pharmacist for home-living multimorbid older people in primary care.

**Methods:**

Participatory action research method, including the active involvement of healthcare professionals, was utilised to develop the PCCM in a public health centre in Finland. The data comprised interview transcripts, workshop materials, field notes, surveys, and memos and were analysed using inductive content analysis.

**Results:**

The PCCM was developed in iterative phases, including planning, acting, observing, and reflecting. The PCCM comprised: 1) A self-management evaluation questionnaire sent before a home visit; 2) A person-centred patient interview at home with a named nurse and a pharmacist; 3) A nurse-led health review and a pharmacist-led clinical medication review; 4) An interprofessional (a GP, a pharmacist and a named nurse) case conference meeting; 5) A care plan, including health and medication plans; and 6) Health support and empowerment interventions. The PCCM shifted working practices in the health centre from *parallel and consultative practice* towards *interprofessional people-centred practice* and more holistic care. The patient's active involvement in their own care was encouraged. Healthcare professionals appreciated the advantages of the new skill-mix, including the clinically trained pharmacist. Building trust among healthcare professionals and between the professionals and the patients was essential.

**Conclusion:**

The successfully developed PCCM improved holistic and more *people-centred care* in primary care. Healthcare professionals appreciated the advantages of the skill mix and found that trust was essential for implementing the PCCM.

## 1 Introduction

While greater population longevity is a triumph for public health, the incidence and prevalence of age-related diseases increase as people live longer. The proportion and number of older people with multiple chronic conditions and complex health needs are growing rapidly over the coming decades. Multimorbidity significantly affects disability, quality of life, and healthcare utilisation.^1^ More effective and holistic patient care models that encourage patients to take an active role in decisions concerning their own health care are needed to delay and prevent functional decline and care dependency.^2^

Indeed, it has been widely suggested that the focus on chronic and multimorbid patients' care should progress from monitoring and supporting medication-taking and health-related behaviour (i.e., adherence) to concordance and further to active patient empowerment.^3^, ^4^, ^5^, ^6^, ^7^, ^8^ According to the WHO (2015),^9^ instead of trying to manage numerous diseases in a siloed approach, the emphasis should be on integrated and people-centred practice, in which health services are organised around the health needs and expectations of people and provided in partnership with them.

While it is well known that multimorbid older people with polypharmacy are at risk of drug-related problems and decreased adherence to medications, irrational prescribing and use of medicines is still a major challenge in this group of patients.^10^, 11., 12 Pharmacists have the potential to expand their clinical role in primary care teams by promoting medication adherence and optimising pharmacotherapy and by conducting clinical medication reviews for multimorbid patients.^13^, ^14^, ^15^, ^16^, ^17^ Clinical medication reviews may improve drug-related outcomes, for example, by improving adherence to medicines and reducing the number of drugs and drug-related problems.^18^, ^19^, ^20^, ^21^, ^22^ Furthermore, community pharmacist-led medication reviews have shown potential to improve certain clinical and healthcare utilisation outcomes in patients with long-term conditions.^23^ However, pharmacists' clinical skills are often underutilised in primary care, and collaboration between public healthcare and community pharmacies is still limited.^24^, ^25^, ^26^

This study aimed to develop an interprofessional people-centred care model (PCCM), including the contribution of a clinically trained pharmacist for home-living older people with multimorbidities in a primary care setting.

## 2 Methods

### 2.1 Setting

In Finland, the healthcare system is public, which is complemented by private and occupational services.^27^ Everyone residing in the country is entitled to adequate health services, and the municipalities are responsible for financing primary and specialised care in the system. Primary care is organised by one or several municipalities together and is usually provided in health centres. Specialist care is organised by regional federations of municipalities called hospital districts.

Municipalities and hospital districts may have their own hospital pharmacies and medicine dispensaries that can dispense drugs to the wards and departments of the hospitals or health centres. Hospital and health centre pharmacists have traditionally provided logistic services, but pharmacists' involvement in patient care and clinical pharmacy services has become more common in Finnish hospitals.^28^ Outpatient medicines are dispensed by privately owned community pharmacies and university pharmacies.^29^ Pharmacists have a legal duty to counsel patients on both the use of prescription and non-prescription drugs. In addition to the statutory services, pharmacies may provide automated dose-dispensing, medication reviews, and consultation services to nursing homes to promote medicines' safe and rational use.

A people-centred care model (PCCM) was developed in a primary care setting in Tornio (a town of 22,000 inhabitants), Finland, for a randomized controlled trial (RCT, Care Plan 2100), between 2013—2018. In the RCT, the aim was to evaluate effectiveness, quality of life, physical performance, and cost-utility of the PCCM in primary care compared with that of usual care.^30^ The study was conducted in close collaboration with Tornio health centre, the only public health centre in Tornio (setting and intervention providers, general practitioners (GPs), nurses, and a pharmacist), Alatornio community pharmacy (intervention provider and a pharmacist), and the pharmacy faculty at the University of Helsinki (intervention providers, researchers, and three pharmacists). The study protocol was approved by the regional ethics committee (North Ostrobothnia Hospital District ethics committee, 32/2014) and registered in the ISRCTN registry (ISRCTN89081244).

### 2.2 Background

Before the start of the research and development project at the Tornio health centre, older people's care and care planning were accessed “traditionally” (i.e., care through an appointment system was mostly accessed by the patient on their own initiative unless a chronic illness, such as asthma or a severe heart condition requiring monitoring, existed). A healthcare professional assessed the patient's needs and usually wrote a care plan with little or no input from the patient or their representative. Person-centred care planning based on each person's preferences did not exist. Furthermore, care within the existing public health system neither included in-depth clinical medication and health reviews, care plans completed in team meetings, nor care coordination by a named healthcare professional.

Basic interprofessional collaboration, especially between GPs and nurses, existed but without coordinated interdisciplinary teams. Mostly healthcare was practised in parallel, employing a consultative model.^31^ It could be argued that the ideal skills-mix^32^^,^^33^ required for multimorbid older people's care planning was not utilised at the health centre.

### 2.3 Participants

The core team of the study included three healthcare professionals working at the health centre (a named nurse, a pharmacist, and an IT-system nurse), a community pharmacist from the local community pharmacy, and three researchers (pharmacists, pharmacy faculty, University of Helsinki). The community pharmacist worked on the project one week per month for one year. The core team members were responsible for organising research activities in the health centre and were the main intervention providers.

The participants were healthcare professionals (GPs, nurses, and a pharmacist) working for the Tornio health centre and the community pharmacist. The model's development process and how action research would benefit the development were discussed with the participants, who were assured about the confidentiality of the research.

The chief physician of the health centre invited the GPs, the nurses, and the pharmacist to develop the PCCM. At all phases, in advance, the researchers informed the participants of every task; they were allowed to opt-out if not consenting to participate.

The research team had a background in clinical pharmacy, pharmacy practice, and pharmaceutical sciences; two with PhDs and one with an MSc. The researchers had dual roles: both researchers and facilitators of, and participants in, the PCCM development and evaluation. The researchers' role as facilitator was to design, plan, and guide the care model development process, and allow open discussion between all participants.

The patients' voice was relayed through the randomized controlled trial: 831 recruitment letters were sent between 2014 and 2016, and 323 patients (39%) returned the recruitment letter. One hundred seventy-four patients were randomized to the intervention group, and of these, 150 received the intervention. At baseline, the mean age of the patients was 81 years, and 62% were women.^30^

### 2.4 Design

The participatory action research method, including the active involvement of healthcare professionals and researchers and comprising five phases (Supplementary Table 1), was utilised to develop, implement, and evaluate the PCCM.^34^^,^^35^ The rationale for using participatory action research was to ensure the involvement and engagement of the participating healthcare professionals in an iterative approach to develop the model for practice and sustain its utilisation after the completion of the RCT. The iterative action research process can be divided into phases: planning, acting, observing, and reflecting.^36^ In this study, preliminary planning took place in phase 1, further planning in phase 2, while the new model was developed and piloted in phase 3 (acting and observing and reflecting Supplementary Table 1). The PCCM was implemented and evaluated in phases 4 (acting) and 5 (observing and reflecting) in a circular way, which led to the model's continuous development, finally resulting in the proposed PCCM. The Standards for Reporting Qualitative Research (SRQR) were applied to the design and reporting of the study.^37^

### 2.5 Data collection and analysis

The study process involved five phases during which the data were collected for developing and evaluating the model (Supplementary Table 1, Supplementary Table 2). Semi-structured interviews, workshops, and structured surveys were developed based on the literature, findings of the previous phases of the study, and discussions within the research team. The researchers arranged meetings, interviews, and workshops with the core team and the participants to develop, implement, and evaluate the PCCM. Field notes, interview transcripts, workshop materials, surveys, and internet-based application conference call memos were collected.

Planning meetings with the core team were organised and held during the RCT between 2014 and 2018. The patient voice was relayed through the clinical health and medication interviews within the RCT^30^^,^^38^; the patients were followed individually for two years between 2014 and 2018 while contributing to the development of the PCCM. Observations of and need for the PCCM development were discussed regularly, and changes were made based on the findings.

Data analysis took place throughout the development, implementation, and evaluation process of the PCCM. All interviews were recorded and transcribed verbatim by the researcher (HE). The data were managed and coded using a qualitative data software programme Atlas.ti [v.8]. The data were analysed (HE) using inductive content analysis to identify analytical categories as they emerge from the data.^39^

## 3 Results

### 3.1 The stages of the people-centred care model (PCCM)

**Fig. 1:**
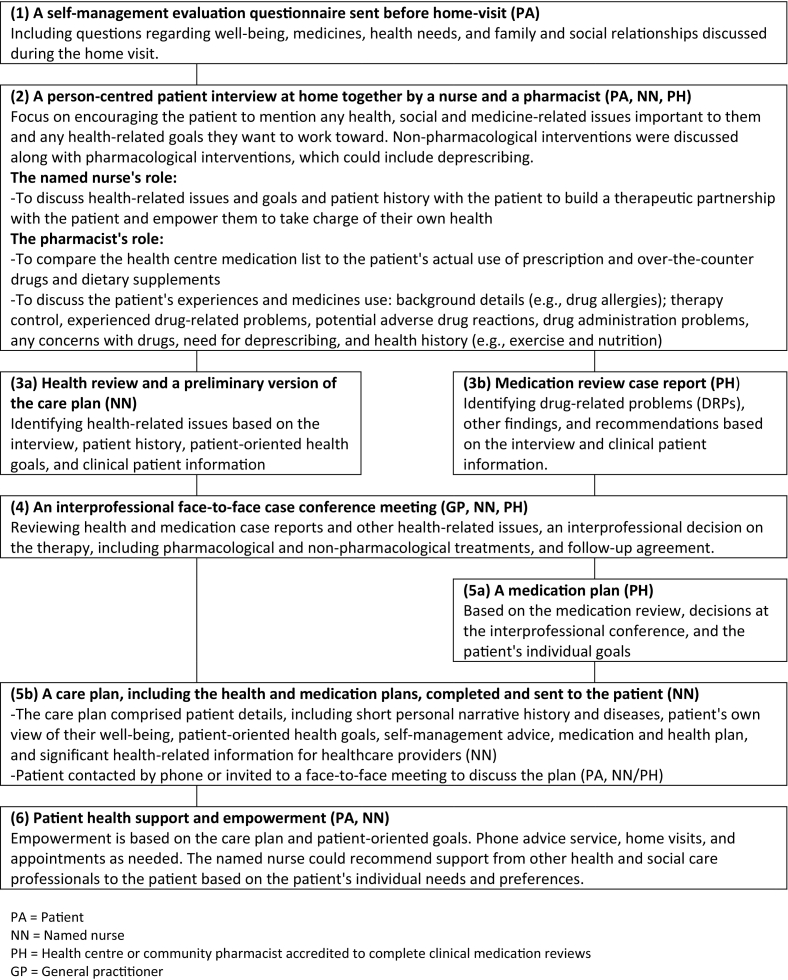
The stages of the people-centred care model (PCCM) for primary care.

The PCCM was developed using the participatory action research method in a primary care health centre (Supplementary Table 1). The final version of the interprofessional PCCM (Fig. 1) comprised six main stages: (1) A self-management evaluation questionnaire sent before home-visit; (2) A person-centred patient interview at home together by a nurse and a pharmacist; (3a and 3b) A nurse-led health review and a pharmacist-led clinical medication review with recommendations; (4) An interprofessional face-to-face (a GP, a pharmacist and a named nurse) case conference meeting; (5a and 5b) A care plan including health and medication plans; and (6) Patient health support and empowerment interventions tailored to the individual needs of the patient and delivered by the named nurse.

### 3.2 Evaluation of the people-centred care model

**Fig. 2:**
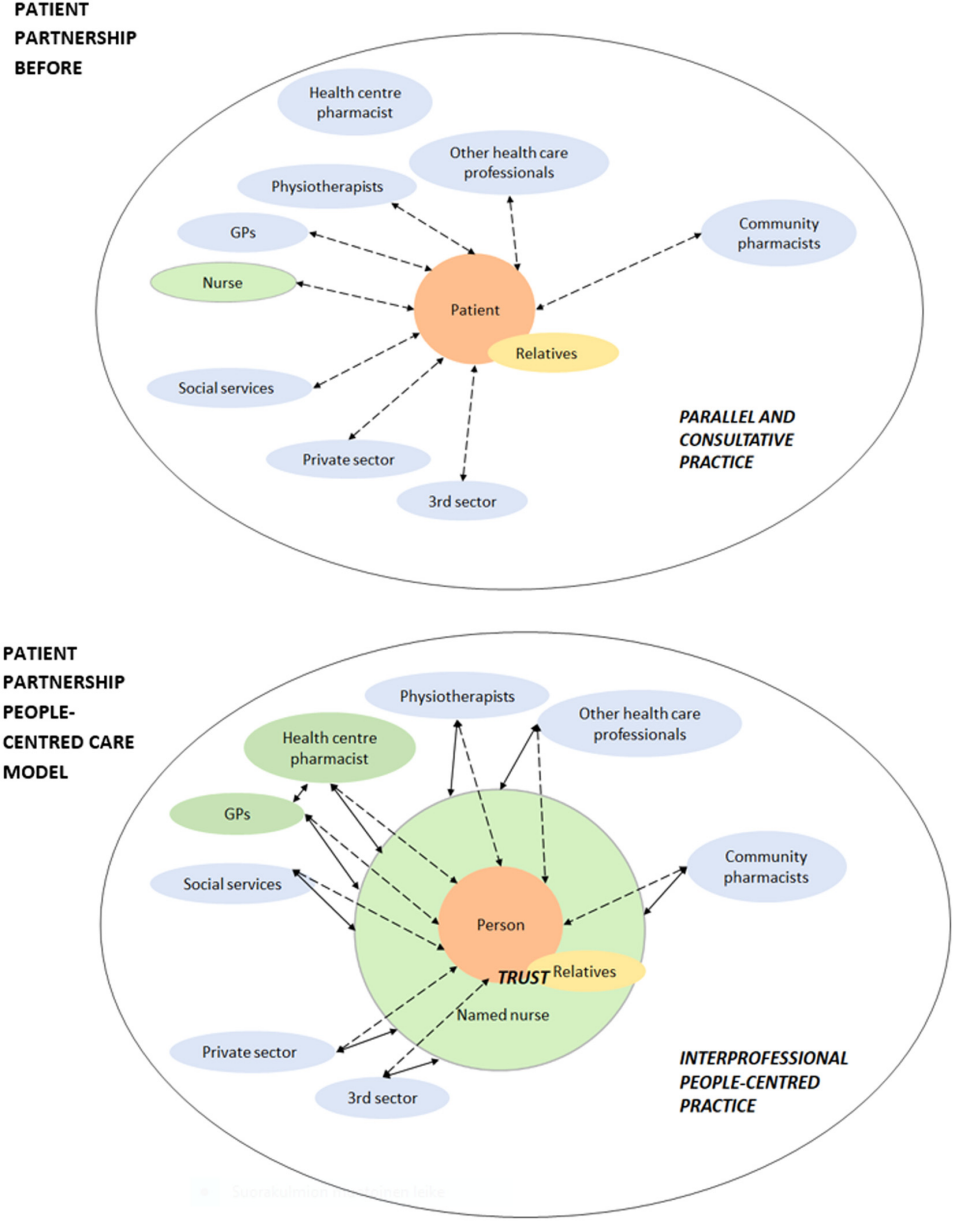
Patient partnership before (passive patient) and after (active patient or person) implementing the people-centred care model. The developed people-centred care model included collaboration in an interprofessional team: the named nurse (a patient advocate), the GPs, and a health centre pharmacist with a new clinical role. The named nurse can recommend support from other health and social care professionals based on the patient's individual preferences recorded in the care plan.

The research team and the participants considered that with the support of the PCCM, the partnership between patients and healthcare professionals had improved in comparison with the situation before the study. The participants emphasised that nearly all patients appeared to be satisfied with the care provided. One patient had said, “Oh, you do take such good care about us now.” Fig. 2 represents patient partnerships or the relationships of a patient, or rather a person, with healthcare professionals before (parallel and consultative practice) and after (interprofessional people-centred practice) the implementation of the PCCM.

A change from system-, disease- and healthcare professional-centred care had occurred to more people-centred care. In the PCCM, the active patient is in the centre; healthcare professionals, especially a named nurse, support and empower the patient. The named nurse can also recommend support from other health and social care professionals to the patient based on the patient's individual needs and preferences recorded in the care plan. The participants found building trust among healthcare professionals and between the professionals and the patients essential for implementing the model. Healthcare professionals may offer support and empowerment for patients, but the most important thing is that people take an active role in their own healthcare:

*“The client has to be active in her/his own care, because s/he is the one who is living her/his own life, and just sees us, the healthcare providers, only for a short period of time in her/his life. Most of the year s/he takes care of herself/himself”.*

Interview, named nurse 1

However, not everyone might easily adapt to the new active role. In the RCT, the mean age of the patients was 81 years.^30^ Thus, the core team thought that, in the future, the care model could be introduced earlier, for example, to newly retired people aged 65 years and above.

### 3.3 Development of the professional roles and interprofessional collaboration

The participants talked about healthcare professionals' roles and what the roles should ideally be in the PCCM (Fig. 1 and Table 1). They perceived that the role of the GP was to be responsible for taking the medical history, providing patient care, and being the medical and clinical expert. The new emergent role of the health centre pharmacist included medication reconciliation, medication reviews, creating medication plans, and providing other clinical pharmacy services. The participants thought that role of the pharmacist had become more clinical than before. The role of the named nurse was essential as a care coordinator and patient advocate, who contributes to identifying the needs of the patient and completes the care plan together with the patient. The named nurse needs to have clinical skills, good social skills, a person-centred holistic attitude, and an open-minded and flexible working style. The named nurse perceived that it had taken him about a year to adopt the new role. The participants noticed the value of the named nurse as a safety net for older people, and especially for those who did not have relatives or whose relatives did not live near them:

**Table 1:** Changes in the professional roles of the healthcare professionals involved in developing and implementing the people-centred care model (PCCM).

Healthcare professional	Role in the traditional model of providing patient care (parallel and consultative practice)	Role in the PCCM of providing patient care (interprofessional people-centred practice)	
Nurse	Takes care of regular follow-up visits (e.g., INR monitoring) and acute care. Multiple nurses work in collaboration with the same patient.	Instead of the traditional role, *the named nurse* works as a care coordinator and patient advocate who identifies the patient's needs together with the patient and creates a care plan. An essential role is to empower the patient to take a more active role in their own health to maintain their quality of life and physical performance. Empowering tools include discussing, increasing knowledge, sharing decision-making, suggesting activities, and assisting lonely persons in connecting with others.	Members of the interprofessional team at the health centre.
General practitioner (GP)	Treats acute and chronic illnesses and refers patients to other healthcare professionals, e.g., specialists in hospitals. No “named GP” system.	In addition to the traditional role, patient care is based on a patient-oriented care plan and keeping medication records up to date—interprofessional teamwork.	
Health centre pharmacist	Responsible for the logistics of medicines in the health centre. No patient contact.	In addition to the traditional role at the health centre, performs at-home patient interviews with medication reconciliation, patient counselling, and clinical medication reviews. Identifies the patient's medication-related needs together with the patient and creates a medication plan. Supports other healthcare professionals in medication-related information needs.	
Community pharmacist	Dispenses medicines and counsels patients in the community pharmacy with little contact with other healthcare professionals.	In addition to the traditional role at the community pharmacy, performs at-home patient interviews with medication reconciliation, patient counselling, and clinical medication reviews. Collaboration between community pharmacies and health centres is improved.	A part-time member of the interprofessional team at the health centre.

*“And [for] these people, whose relatives do not live near them, so this kind of a safety net with the named nurse, it is a safety net also for the relatives, many of them feel, that it is lovely that there is someone they can call.”*

Interview, GP 3

The PCCM benefits healthcare professionals; other nurses in the health centre had started to work similarly to the named nurse, which was expected according to the theory of diffusion of innovation.^40^ The participants described that collaborative working during the project involved and increased learning from each other, information sharing, and critical thinking about their roles:

*“ Yes, I think that it has been great in that way, it has been educational also for a doctor, because [we have had] […] the specialist in pharmaceutical care, and [when] questions have been asked about drug treatments and [their] indications and possible [drug related] problems, which the pharmacist has identified. So, yes, in that way, it is very informative for a doctor, too.”*

Interview, GP 1

Another GP talked about obtaining a more detailed medical history through teamwork:

*“ And then there is this, what has changed in the professional role, so maybe [the patient's] medical history has become more precise. Because now, when one has been astonished by what the pharmacist and the nurse have told us about the home visits: that there is this and that in the cupboards. So these [patients] don't necessarily tell [those things] here [at the GPs]. Then we are surprised about [their] kidney function, and surprised about other things. So, yes, a more precise medical history that is something that has become maybe a bit more precise.”*

Interview, GP 3

One of the GPs had noticed that the skills of the health centre pharmacist had been underutilised, and the skills mix among healthcare professionals could be improved:

*“ Maybe we have not utilised all the potential the pharmacist has.”*

Interview, GP 3

The health centre pharmacist had had a logistical role; now, she had a more clinical and patient-focused role and found this new role rewarding. Differences in the professional roles in the traditional care model and PCCM are described in Table 1.

### 3.4 The requirements and advantages of the PCCM

**Fig. 3:**
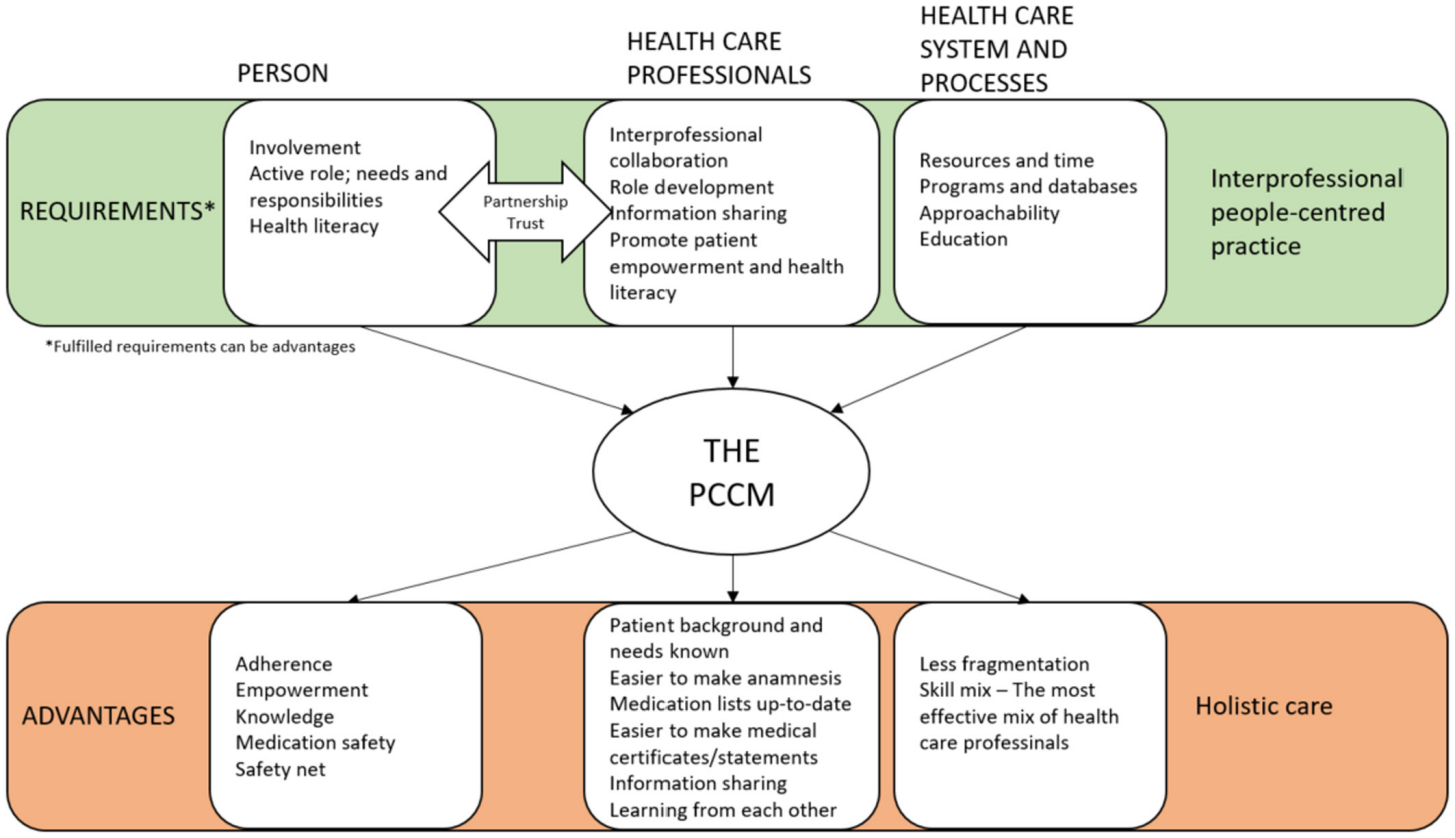
Requirements for, and advantages of, the people-centred care model (PCCM).

The participants identified multiple requirements for and advantages of the PCCM for the patient, healthcare professionals, and healthcare systems (Fig. 3). They found that health conditions, medications used, and health-related goals were better identified for those patients who had been involved in the project. Improving patient partnerships, patient-oriented health goal settings, and patients' active participation in making care plans, required both patients' and healthcare professionals' role and trust development and strong interpersonal and interprofessional collaboration. Fulfilled requirements can also be an advantage. The participants agreed that the most important advantage of the PCCM is its potential to offer more holistic patient care within primary care. However, the participants also emphasised that it is important to identify those who might benefit the most from this kind of holistic care due to limited resources for healthcare services. Furthermore, it was discussed that not all patients might want or need this kind of service and might require further convincing of its advantages before the model becomes expected practice by everyone.

## 4 Discussion

This paper reports the development and implementation of the people-centred care model (PCCM) for home-living older people with multimorbidities in a primary care health centre. The PCCM is a process in which patients and healthcare professionals collaborate, discuss, and agree on an action plan to achieve person-oriented goals that are most relevant to the patient. The participants found that successfully implemented PCCM improved interprofessional collaboration in care planning and healthcare professionals appreciated the advantages of the skill mix. The PCCM has its roots in the Chronic Care Model,^5^^,^^6^^,^^41^^,^^42^ and it was developed, implemented, and evaluated interprofessionally with the healthcare providers, the participants, and the researchers utilising action research in a health centre.

Interprofessional teamwork is an essential element of the PCCM. The core of the PCCM interprofessional team was composed of a triad (a GP, a nurse, and a pharmacist) working closely together. Improving interprofessional teamwork is essential to respond to the increasingly complex health needs of the patients and develop, promote, and maintain population health and provide holistic care.^43^^,^^44^ Interprofessional collaboration involves regular negotiation and interaction between healthcare professionals, which values the expertise and contributions that various professionals bring to patient care.^45^

In this study, collaboration among the healthcare professionals fostered their awareness of each other's knowledge and skills. In addition, numerous previous studies have shown that trust between healthcare professionals is important in interprofessional collaboration,^46^, ^47^, ^48^ which also emerged from our data. The successful shift from parallel and consultative practice towards interprofessional people-centred practice in the PCCM was lengthy and not easy for the participants. Yet, the organisational culture in Tornio was rather flexible. Healthcare professionals adopted and adapted to the developed model, which might be due to the small size of the health centre and participation in previous developmental projects.

In the PCCM, the role of the named nurse is to work as a care coordinator and patient advocate and to support and empower person-oriented changes in patient behaviour that may result from patients being more involved in their own care and maintaining their quality of life and physical performance. This central role is often performed by nurses to improve coordination and continuity of care, especially for patients with complex care needs.^49^ It was found that with experience and professional growth, the named nurse's working methods evolved to become even more person-oriented. However, the adoption of the working method took time.

In the PCCM, the pharmacist's role changed from a traditional logistical role still commonly prevalent in Finnish healthcare settings at the time of the study to a more clinical role, which was noticed and valued by the participants. While the role of the pharmacist has traditionally been associated with ordering, stocking, and dispensing medicines in health centres with no direct patient contact, extended clinical pharmacist roles and pharmaceutical care involve more patient-centred services, such as improving health by reducing drug-related problems, promoting better medication adherence, and conducting medication reviews.^28^^,^^50^^,^^51^ This development is not new; Hepler & Strand introduced the concept of pharmaceutical care more than 30 years ago,^52^ and, for example, NICE's (National Institute for Health and Care Excellence) medicines optimisation framework is defined as ‘a person-centred approach to safe and effective medicines use, to ensure people obtain the best possible outcomes from their medicines’.^53^ Indeed, pharmacists are enthusiastic and desiring an extended and more patient-centred role and interdisciplinary teamwork in primary care.^24^^,^^54^ However, some barriers have been experienced during pharmacist integration into primary care teams.^55^ These include a lack of role definition, limited support and mentorship, and a lack of adequate resources. In our study, the pharmacists in the health centre and the community pharmacy were supported and mentored by the researchers, whose expertise was in clinical pharmacy. Nevertheless, adopting a new, more clinical role was challenging for healthcare professionals.

The essential features of the PCCM are a pharmacist-led clinical medication review and a nurse-led health review with an at-home patient interview focusing on supporting the patient in identifying their needs, an interprofessional case conference meeting, a comprehensive care plan, and patient health support and empowerment interventions delivered by the named nurse.^38^ This kind of care model is in line with the WHO (2015) statement,^9^ which says it is important to empower people to take charge of their own health rather than being passive recipients of services. The disease outcome-based paradigm in healthcare is so strong that it might be the most important barrier to goal-oriented care.^56^ Healthcare culture has traditionally valued managing each disease according to guidelines and population goals above the patient-oriented needs. The PCCM's care plans were based on person-oriented goal-setting and were composed in a shared decision-making process.^57^ In addition, patients' medicines were optimised, which means a person-focused approach to ensuring that the right patients get the right choice of medicines, or choice of non-medical care or treatment when applicable, at the right time, and that they get the best outcomes from their medicines.^58^ Indeed, the participants agreed that patient care was more holistic after adopting the PCCM than before in the traditional style of patient care. In addition, the PCCM dominated usual care since it was more effective and less costly in an RCT-based cost-utility analysis reported in another research article by Kari et al..^30^

There are strengths and limitations to our study. While the patient voice was relayed through the clinical health and medication interviews,^38^ the patients were not direct participants of this qualitative study. In future studies and development projects, patients should be more directly involved and part of the initial designing, co-creation, implementation and evaluation phases so that the possible benefits that interventions generate are captured, and weaknesses of the models are better identified.^59^ The study's strength is the utilisation of an iterative participatory action research method, including data collection and analysis that took place throughout the development, implementation, and evaluation process of the PCCM, improving the model in each cycle.^60^ The data were analysed using inductive content analysis, and quotations were used to indicate the trustworthiness of results and to reflect the participants' voice,^61^ but the researchers' background and values influence the interpretation of the results and how they are reported. Our findings and the PCCM could be transferable to other contexts or settings. However, it has to be considered that every action research project and its participants are unique.

## 5 Conclusion

An interprofessional people-centred care model was successfully developed between patients and healthcare professionals in a primary care setting. The named nurse was the key professional to identify person-oriented goals that were most relevant to the patient, provide care coordination, and empower the patient. The GPs and nurses appreciated the pharmacist's new, more clinical role. Overall, interprofessional collaboration in care planning and medicine optimisation was improved, and healthcare professionals appreciated the advantages of the skill mix. Trust among healthcare professionals and between the professionals and the patients was essential for implementing the PCCM.

## Funding

This work was supported by Vappu and Oskari Yli-Perttula Foundation, Tornio city, and The Association of the Finnish Pharmacies (AFP). The researchers were independent of the funders.

## Declaration of Competing Interest

The authors declare that they have no known competing financial interests or personal relationships that could have appeared to influence the work reported in this paper.

## Appendix A Supplementary data

Supplementary material Image 1

## References

[1] Marengoni A., Angleman S., Melis R. (2011). Aging with multimorbidity: a systematic review of the literature. Ageing Res Rev.

[2] WHO World Health Organization (2017).

[3] Araujo de Carvalho I., Epping-Jordan J., Pot A.M. (2017). Organizing integrated health-care services to meet older people’s needs. Bull World Health Organ.

[4] Bell J.S., Airaksinen M.S., Lyles A., Chen T.F., Aslani P. (2007). Concordance is not synonymous with compliance or adherence. Br J Clin Pharmacol.

[5] Bodenheimer T., Wagner E.H., Grumbach K. (2002). Improving primary care for patients with chronic illness. JAMA..

[6] Bodenheimer T., Wagner E.H., Grumbach K. (2002). Improving primary care for patients with chronic illness: the chronic care model, part 2. JAMA..

[7] Cloninger C.R. (2011). Person-centred integrative care. J Eval Clin Pract.

[8] Pulvirenti M., McMillan J., Lawn S. (2014). Empowerment, patient centred care and self-management. Health Expect.

[9] World Health Organization (WHO) (2015). WHO Document Production Services.

[10] Kaufmann C.P., Stämpfli D., Hersberger K.E. (2015). Determination of risk factors for drug-related problems: a multidisciplinary triangulation process. BMJ Open.

[11] WHO, World Health Organization (2017).

[12] Plácido A.I., Herdeiro M.T., Morgado M., Figueiras A., Roque F. (2020). Drug-related problems in home-dwelling older adults: a systematic review. Clin Ther.

[13] Laaksonen R., Duggan C., Bates I. (2010). Performance of community pharmacists in providing clinical medication reviews. Ann Pharmacother.

[14] Blenkinsopp A., Bond C., Raynor D.K. (2012). Medication reviews. Br J Clin Pharmacol.

[15] Leikola S., Tuomainen L., Peura S. (2012). Comprehensive medication review: development of a collaborative procedure. Int J Clin Pharmacol Ther.

[16] Bulajeva A., Labberton L., Leikola S. (2014). Medication review practices in European countries. Res Soc Adm Pharm.

[17] Kiiski A., Airaksinen M., Mäntylä A. (2019). An inventory of collaborative medication reviews for older adults - evolution of practices. BMC Geriatr.

[18] Holland R., Desborough J., Goodyer L., Hall S., Wright D., Loke Y.K. (2008). Does pharmacist-led medication review help to reduce hospital admissions and deaths in older people? A systematic review and meta-analysis. Br J Clin Pharmacol.

[19] Lenander C., Elfsson B., Danielsson B., Midlov P., Hasselstrom J. (2014). Effects of a pharmacist-led structured medication review in primary care on drug-related problems and hospital admission rates: a randomized controlled trial. Scand J Prim Health Care.

[20] Huiskes V.J., Burger D.M., van den Ende C.H., van den Bemt B.J. (2017). Effectiveness of medication review: a systematic review and meta-analysis of randomized controlled trials. BMC Fam Pract.

[21] Jokanovic N., Tan E.C., Sudhakaran S. (2017). Pharmacist-led medication review in community settings: an overview of systematic reviews. Res Soc Adm Pharm.

[22] Vinks T.H., Egberts T.C., de Lange T.M., de Koning F.H. (2009). Pharmacist-based medication review reduces potential drug-related problems in the elderly: the SMOG controlled trial. Drugs Aging.

[23] Al-Babtain B., Cheema E., Hadi M.A. (2021 Apr 29). Impact of community-pharmacist-led medication review programmes on patient outcomes: a systematic review and meta-analysis of randomised controlled trials. Res Soc Adm Pharm.

[24] Mossialos E., Courtin E., Naci H. (2015). From “retailers” to health care providers: transforming the role of community pharmacists in chronic disease management. Health Pol.

[25] Wertheimer A.I. (2018). The underutilised pharmacist. J Pharm Health Serv Res.

[26] Seston E.M., Schafheutle E.I., Willis S.C. (2021 Nov 22). “A little bit more looking…listening and feeling” a qualitative interview study exploring advanced clinical practice in primary care and community pharmacy. Int J Clin Pharm.

[27] Keskimäki I., Tynkkynen L.K., Reissell E. (2019). Finland: health system review. Health Syst Transi.

[28] Schepel L., Aronpuro A., Kvarnström K. (2019). Strategies for improving medication safety in hospitals: evolution of clinical pharmacy services. Res Soc Adm Pharm.

[29] Airaksinen M., Toivo T., Jokinen L. (2021). Policy and vision for community pharmacies in Finland: a roadmap towards enhanced integration and reduced costs. Pharm Pract (Granada).

[30] Kari H., Äijö-Jensen N., Kortejärvi H. (2021). Effectiveness and cost-effectiveness of a people-centred care model for community-living older people versus usual care – a randomised controlled trial. Res Soc Adm Pharm.

[31] Boon H., Verhoef M., O’Hara D., Findlay B. (2004). From parallel practice to integrative health care: a conceptual framework. BMC Health Serv Res.

[32] Buchan J., Dal Poz M.R. (2002). Skill mix in the health care workforce: reviewing the evidence. Bull World Health Organ.

[33] The World Health Report (2000).

[34] Baum F., MacDougall C., Smith D. (2006). Participatory action research. J Epidemiol Community Health.

[35] Hampshire A.J. (2000). What is action research and can it promote change in primary care?. J Eval Clin Pract.

[36] Koshy E., Koshy V., Waterman H. (2011).

[37] O’Brien B.C., Harris I.B., Beckman T.J., Reed D.A., Cook D.A. (2014). Standards for reporting qualitative research: a synthesis of recommendations. Acad Med.

[38] Kari H., Kortejärvi H., Airaksinen M., Laaksonen R. (2018). Patient involvement is essential in identifying drug-related problems. Br J Clin Pharmacol.

[39] Pope C., Ziebland S., Mays N. (2000). Qualitative research in health care. Analysing qualitative data. BMJ.

[40] Rogers E.M. (2013).

[41] Wagner E.H., Austin B.T., Von Korff M. (1996). Organizing care for patients with chronic illness. Milbank Q.

[42] Wagner E.H. (1998). Chronic disease management: what will it take to improve care for chronic illness?. Eff Clin Pract.

[43] Samuelson M., Tedeschi P., Aarendonk D., de la Cuesta C., Groenewegen P. (2012). Improving interprofessional collaboration in primary care: position paper of the European forum for primary care. Qual Prim Care.

[44] Xyrichis A., Lowton K. (2008). What fosters or prevents interprofessional teamworking in primary and community care? A literature review. Int J Nurs Stud.

[45] Reeves S., Pelone F., Harrison R., Goldman J., Zwarenstein M. (2017). Interprofessional collaboration to improve professional practice and healthcare outcomes. Cochrane Database Syst Rev.

[46] Bardet J.-D., Vo T.-H., Bedouch P., Allenet B. (2015). Physicians and community pharmacists collaboration in primary care: a review of specific models. Res Soc Adm Pharm.

[47] Löffler C., Koudmani C., Böhmer F. (2017). Perceptions of interprofessional collaboration of general practitioners and community pharmacists – a qualitative study. BMC Health Serv Res.

[48] Makowsky M.J., Schindel T.J., Rosenthal M. (2009). Collaboration between pharmacists, physicians and nurse practitioners: a qualitative investigation of working relationships in the inpatient medical setting. J Interprof Care.

[49] Saint-Pierre C., Herskovic V., Sepulveda M. (2018). Multidisciplinary collaboration in primary care: a systematic review. Fam Pract.

[50] European Association of Hospital Pharmacists (2020). The European statements of hospital pharmacy. Eur J Hosp Pharm.

[51] Franklin B.D., van Mil J.W.F. (2005). Defining clinical pharmacy and pharmaceutical care. Pharm World Sci.

[52] Hepler C.D., Strand L.M. (1990). Opportunities and responsibilities in pharmaceutical care. Am J Hosp Pharm.

[53] NICE, National Institute for Health and Care Excellence (2016). Quality standard [QS120].

[54] Butterworth J., Sansom A., Sims L., Healey M., Kingsland E., Campbell J. (2017). Pharmacists’ perceptions of their emerging general practice roles in UK primary care: a qualitative interview study. Br J Gen Pract.

[55] Jorgenson D., Laubscher T., Lyons B., Palmer R. (2014). Integrating pharmacists into primary care teams: barriers and facilitators. Int J Pharm Pract.

[56] Reuben D.M., Tinetti M.E. (2012). Goal-oriented patient care – an alternative health outcomes paradigm. N Engl J Med.

[57] Elwyn G., Frosch D., Thomson R. (2012). Shared decision making: a model for clinical practice. J Gen Intern Med.

[58] Royal Pharmaceutical Society (2013). Medicines Optimisation: Helping Patients to Make the Most of Medicines. https://www.rpharms.com/Portals/0/RPS%20document%20library/Open%20access/Policy/helping-patients-make-the-most-of-their-medicines.pdf%3e.

[59] McCarron T.L., Clement F., Rasiah J. (2021). Patients as partners in health research: a scoping review. Health Expect.

[60] Saramunee K. (2022). Applying action research in pharmacy practice. Res Soc Adm Pharm.

[61] Elo S., Kääriäinen M., Kanste O., Pölkki T., Utriainen K., Kyngäs H. (2014).

