# Supplementary material for: Developing an interprofessional people-centred care model for home-living older people with multimorbidities in a primary care health centre: A community-based study
Source: Explor Res Clin Soc Pharm. 2022 Feb 5;5:100114. doi: 10.1016/j.rcsop.2022.100114 (PMC9030719; doi:10.1016/j.rcsop.2022.100114)
Supplement: Supplementary file 1 — Supplementary material [file mmc1.docx]

**Supplementary Table 1. The description of the phases of the study in developing the people-centred care model (PCCM), using the participatory action research method.**

| **Study phase** | **Time period** | **Description of study phases in developing the PCCM.** |
| --- | --- | --- |
| 1 | 08/2013–08/2014 | **Preliminary planning of the research project (Planning)**  A preliminary version of the PCCM was based on a literature review of the national and international care model frameworks, such as the Chronic Care Model (CCM) [5–6, 41–42], an organisational approach for chronic care management in primary care settings. The CCM advocates a change from the traditional acute model of primary healthcare to a model that addresses a collaborative, person-centred approach to improve chronic disease management. Other important care model was the Finnish National Institute for Health and Welfare Potku2 chronic care model development project [62]. To familiarise themselves with the Finnish Potku2 care model, the principal investigator and the named nurse visited two health centres actively participating in the development project. Preliminary versions of the PCCM and the research protocol were also introduced and discussed with six general practitioners (GPs).  While *the research team* evaluated the elements of the previous care models and frameworks, they also reviewed literature; none of the care models was considered person-centred enough, effectiveness and cost-effectiveness studies were scarce, and long-term effects were unknown for the aged in primary care. Additionally, these previous care models did not involve clinical medication reviews. Based on these observations, *the research team* decided to develop a person-centered PCCM that involves pharmacist-led clinical medication reviews. The overarching aim of the PCCM was to recognise and treat each patient as a person, encourage their active role in collaborative health goal-setting, empower multimorbid patients to live well with long-term conditions and develop a partnership between patients and healthcare professionals. |
| 2 | 09/2014 | **Identifying the needs and targets, discussing the current state of care for older people (Planning)**  A focus group interview was conducted to discuss how to care for multimorbid older people at the health centre and to identify how to develop the current system. Additionally, *the research team* introduced a preliminary version of the PCCM to *the participants* to give feedback on the model, and modifications based on their observations could be made. Several themes and issues emerged from the interview data through inductive content analysis: "Patients' medication records are not up-to-date" was the most frequent perception, requiring action. Thus, *the researchers* and *the participants* agreed that clinical medication reviews would be an essential element of the care model, leading to updating the patients' medication records in the health centre. Other identified issues were the software being user-unfriendly in the Healthcare IT systems, information and communication problems between healthcare professionals during the patient pathway, a lack of patient knowledge and education of medications and self-management skills, and strict regulatory environment in the health sector. Additionally, the lack of resources for healthcare was discussed, and some of *the participants* questioned if any new processes could be developed and implemented without additional workforce. *The researchers* explained that by enhancing interprofessional teamwork, defining and developing new roles and responsibilities, supporting patients to manage their own health, and improving care planning, there could be more efficient and people-centred ways to provide care for older people without additional costs or workforce. |
| 3 | 09/2014–03/2015 | **Creating and examining the new model, piloting the model (Acting, observing, and reflecting)**  The first version of the PCCM comprised a pharmacist-led clinical medication review and a nurse-led health review with an at-home patient interview, an interprofessional (a GP, a pharmacist, and a named nurse) case conference meeting, a care plan, and patient health support and empowerment interventions delivered by the named nurse. To support the patients to prepare for the at-home interviews, a self-management evaluation questionnaire, including questions regarding well-being, medicines, health needs, and family and social relationships [63], was sent to them. At the start, there were two separate at-home visits, but to save time and resources, *the participants* suggested after a few visits that both a pharmacist and the named nurse could be present at the same time (second version of the PCCM), which was supported by *the researchers*. Otherwise, *the participants* were concordant that the care model was appropriate to their needs and would also benefit aged multimorbid patients. However, *the researchers* encouraged *the participants* to continuously mention any issues or needs of development after piloting the model. |
| 4–5 | 04/2015-01/2018 | **Implementing and evaluating the PCCM, reflecting on the process, further development of the PCCM (Acting, observing, and reflecting)**  While the second version of the PCCM was implemented, the following key themes emerged from the collected data (field notes, interview transcripts, workshop materials, surveys, photographs, and internet-based conference call memos): *the stages of the PCCM, evaluation of the people-centred care model*, *development of the professional roles and interprofessional collaboration*, and *the requirements and advantages of the PCCM. The researchers'* roles as facilitators of the action research and development project were important throughout all phases.  ***Care model development***  The participants observed an evolution of the focus and execution of the patient interviews during the study*.* In the beginning, the at-home interviews were more structured; the named nurse utilised the primary care clinical records and discussed health-related issues and goals with the patients. The pharmacist compared the health centre medication list to the patient's actual use of prescription and over-the-counter drugs and dietary supplements and discussed the patient's experiences and medicines use: experienced drug-related problems; potential adverse drug reactions; problems with medicine administration; any concerns with medicines; and health history. Later, with experience and potential professional growth, the focus of the interviews evolved to become more to person-oriented health goal setting including pharmacological and non-pharmacological treatments, the building of therapeutic partnership with the person, and empowering them to take charge of their own health and important elements improving quality of life. This transformation from interviewing to health and person-oriented discussions also led to the improvement of the care plan so that the personal history section in the care plan was completed using a more narrative approach.  The final version of the care plan comprised patient details, including short personal history and diseases, patients' own view of their well-being, patient-oriented health goals, self-management advice, medication and health plan, and significant health-related information for healthcare providers. *The participants* also suggested adding a notification within the electronic patient record system to inform the concerned healthcare professionals in the health centre that a PCCM care plan had been completed; this was implemented into the notifications that pop up when electronic patient records are opened. Eventually, the final version of the PCCM was developed based on the findings of all the previous phases of the study (Figure 1). |

[62] A. Hujala, Oksman E. Rijken, H. Taskinen, S. Rissanen. The POTKU project (Potilas kuljettajan paikalle, Putting the Patient in the Driver's Seat), Finland, viewed 22.11.2021, http://www.icare4eu.org/pdf/POTKU_Case_report.pdf

[63] Sosiaali- ja terveydenhuollon kansallinen kehittämisohjelma (KASTE). POTKU2, Omahoitolomake [in Finnish], viewed 14.10.2020, https://www.hel.fi/static/sote/itsehoito/lomakkeet/omahoitolomake_N.pdf

**Supplementary Table 2. Data collection**

| **Study phase** | **Date/**  **Time period** | **Purpose for data collection** | **Participants** | **Collected data** |
| --- | --- | --- | --- | --- |
| **Group and pair interviews** | | | | |
| 2 | 18.9.2014 | Identification of the needs and targets, and discussing the current state of care for older people. Introducing preliminary version of the people-centred care model (PCCM) to the participants | 4 nurses, 1 geriatrician, 1 health centre pharmacist,  1 community pharmacist, 1 IT-system nurse, 3 research pharmacists. (Focus group interview) | Recorded interview |
| 3 | 27.11.2014 | Evaluation of the pilot | 1 health centre pharmacist, 1 named nurse | Recorded interview |
| 4–5 | 11.9.2015 | Evaluation of the care plan and how to further improve it. | 6 general practitioners (GPs), 2 nurses, 1 health centre pharmacist, 1 community pharmacist, 1 director of social and health services in Tornio, 1 researcher | Recorded,  included a workshop. |
| **Workshops** | | | | |
| 4–5 | 23.9.2016 | Evaluation of a role and requirements of a patient, GP, pharmacist, and named nurse in the care model | 10 GPs, 4 nurses, 1 health centre pharmacist, 2 researchers | Written assignments |
| 4–5 | 6.10.2017 | Evaluation of the role of the pharmacist in updating patients' medication records | 11 GPs, 1 health centre pharmacist,  2 researchers | Written assignments |
| **Interviews** | | | | |
| 4–5 | 31.1.2017 and 1.2.2017 | Evaluation of advantages and requirements of the care model. | 3 GPs, 1 named nurse, 1 health centre pharmacist | Recorded interview,  5 interviews |
| **Surveys** | | | | |
| 3 | 30. and 31.10.2014 | Evaluation of the pilot | 1 nurse, 1 community pharmacist | Written comments |
| **Meetings in Tornio** | | | | |
| 4–5 | 23.9.2016 | Evaluation of patient groups that would benefit from the PCCM | 1 named nurse, 1 health centre pharmacist, 2 researchers | Notes, photographs |
| 4–5 | 5.-6.10.2017,  4 meetings | Evaluation of the project and future expectations | 2 GPs, 1 named nurse,  1 research nurse, 2 researchers | Research diary |
| **Internet-based application conference calls** | | | | |
| 4–5 | 16.2.2015-11.1.2018,  9 conference calls | Project analysis and planning | Changing composition of the core team, including participants from Tornio health centre and research team | Memos, research diary |
| **Researcher meetings, phone calls, emails** | | | | |
| 1–5 | 8/2013-12/2017, researcher meetings/phone calls | Planning and evaluating the project and analysing its results | Changing the composition of the researchers from the University of Helsinki Faculty of Pharmacy and other healthcare professionals and researchers. | Research diary, emails |
